# Inflammatory biomarkers and physiological reserve: an explainable machine learning model for predicting postoperative pulmonary complications in elderly laparoscopic surgery

**DOI:** 10.3389/fcimb.2026.1677260

**Published:** 2026-03-06

**Authors:** Di Liu, Fei Jiang, Hui Huang, Yong Yang, Lei Zou, Min Ye

**Affiliations:** 1 Anesthesia Surgery Center, The First People’s Hospital of Neijiang, Neijiang, Sichuan, China; 2 Department of Radiology, The First People’s Hospital of Neijiang, Neijiang, Sichuan, China; 3 Department of Anesthesiology, The First Affiliated Hospital of Chongqing Medical University, Chongqing, China

**Keywords:** early warning, elderly, gradient boosting machine, laparoscopic surgery, machine learning, postoperative pulmonary complications

## Abstract

**Introduction:**

Postoperative pulmonary complications (PPCs) significantly impact the prognosis of elderly patients undergoing laparoscopic surgery, yet reliable tools for early risk stratification are lacking. This study aimed to develop and externally validate a machine learning (ML) model to predict PPCs using preoperative and intraoperative data available at the point of surgical closure.

**Methods:**

A multicenter retrospective cohort study was conducted involving 1,415 elderly patients (age >60 years) from two tertiary hospitals in China. The primary outcome was clinically significant PPCs (Clavien-Dindo Grade ≥ II) within 7 days postoperatively. Nine ML algorithms were trained and optimized using a nested 5-fold cross-validation framework. The Synthetic Minority Over-sampling Technique (SMOTE) and Boruta algorithm were employed to address class imbalance and feature selection, respectively. The model’s performance was evaluated in an internal development cohort and an independent external validation cohort (n=102).

**Results:**

Among the evaluated algorithms, the Gradient Boosting Machine (GBM) demonstrated superior performance, achieving an Area Under the Curve (AUC) of 0.691(95% CI: 0.617-0.762) (Sensitivity 65.2%, Specificity 83.4%) in the internal cohort, Notably, the model exhibited superior performance in the external validation cohort with an AUC: 0.755 (95% CI: 0.652–0.849), indicating excellent generalizability without overfitting. The decision curve analysis confirmed that the GBM model provided a higher net clinical benefit than the default strategies. SHAP (SHapley Additive exPlanations) analysis identified Surgery Duration, Preoperative Albumin, and inflammatory markers (CRP, WBC) as top predictors, reflecting the interplay between surgical stress and physiological reserve. Decision Curve Analysis (DCA) confirmed the model’s clinical utility, showing a net benefit across a threshold probability range of 30%–90%.

**Conclusion:**

The GBM-based dynamic model offers a robust, interpretable, and generalizable tool for the early prediction of PPCs in elderly laparoscopic surgery patients. By enabling risk assessment immediately upon surgical completion, this tool facilitates the shift from reactive treatment to proactive prevention and personalized perioperative management.

## 1 Introduction

Postoperative pulmonary complications (PPCs) are significant contributors to perioperative mortality and morbidity in noncardiac surgical patients (Fernandez-Bustamante et al., 2017; Miskovic and Lumb, 2017; Qaseem et al., 2006). Research indicates that the predictive value of PPCs for long-term mortality in noncardiac patients surpasses that of cardiac complications (Mills, 2018; NIHR Global Health Research Unit on Global Surgery and STARSurg Collaborative, 2024). Despite the perception that laparoscopic surgery minimizes the risk of PPCs due to its minimally invasive nature, elderly patients remain susceptible to PPCs due to various factors such as complications, pneumoperitoneum, unique positioning, postoperative pain, with an incidence rate ranging from 20% to 40% (Pensier et al., 2025), particularly within the initial week post-surgery (Elefterion et al., 2024). PPCs lead to prolonged hospitalization, increased healthcare costs, and a substantial decline in quality of life and long-term survival (Alfirevic et al., 2023; Fernandez-Bustamante et al., 2017; Li et al., 2024; Mills, 2018; Moore et al., 2017; Pensier et al., 2025; Qaseem et al., 2006; Sun et al., 2023). While various risk scores exist, most rely on subjective preoperative assessments and fail to capture the physiological impact of surgical stress. Furthermore, although some studies suggest using postoperative laboratory markers for prediction, these often delay the critical decision-making process during the immediate transition from the operating room to the post-anesthesia care unit (PACU) or intensive care unit (ICU).

Current assessments of PPCs risk primarily utilize traditional tools like ARISCAT scores or logistic regression models. While valuable, these methods have notable limitations: they incorporate a limited range of risk factors and struggle to integrate the complex, multidimensional clinical data generated perioperatively, such as comorbidities in the elderly and intraoperative variables. Furthermore, traditional models are inadequate at managing complex nonlinear relationships and high-dimensional feature interactions, resulting in predictive power (AUC) typically between 0.70 and 0.80, which falls short of clinical risk stratification needs (Peng et al., 2022; Zorrilla-Vaca et al., 2023). In contrast, machine learning models, such as deep neural networks and CNNs, excel in automatic feature extraction and complex pattern recognition for high-dimensional, heterogeneous data. They effectively identify potential risk signals and capture complex nonlinear interactions, demonstrating significant advantages in clinical multi-domain risk prediction (Chau et al., 2025; Kim et al., 2023; Li et al., 2024; Liu et al., 2023; Sagar et al., 2022).

There is a shortage of high-quality research on the utilization of Machine learning (DL) technology for predicting specific risks in elderly patients, a particularly vulnerable group for Postoperative Pulmonary Complications (PPCs), notably in the context of laparoscopic surgery involving general anesthesia and endotracheal intubation (Liu et al., 2023; Yoon et al., 2024). Despite the minimally invasive nature of such surgeries, challenges to the respiratory system persist due to factors like pneumoperitoneum, unique patient positioning, and anesthesia management. Therefore, further comprehensive investigations are warranted to validate the efficacy and clinical significance of DL in this setting. A clinically utility model must provide timely results to guide early intervention. Integrating preoperative baseline characteristics with intraoperative surgical data offers a ‘real-time’ snapshot of a patient’s risk profile at the exact moment of surgical closure. This immediate risk stratification is particularly crucial for elderly patients, whose physiological reserve is limited and who require proactive respiratory management to prevent the onset of PPCs. Therefore, this study aimed to develop and validate a robust machine learning-based model for predicting PPCs in elderly patients undergoing laparoscopic surgery, utilizing only data available up to the point of surgical completion. By focusing on preoperative baseline factors and intraoperative physiological variables, we sought to provide an immediate and objective risk assessment tool. Our objective was to empower clinicians to identify high-risk individuals at the earliest possible stage, enabling the timely implementation of personalized lung-protective strategies and optimized postoperative disposition.

## 2 Section snippets

### 2.1 Study design and population

A retrospective cohort study was carried out on 1,415 elderly patients who underwent laparoscopic surgery with general anesthesia and endotracheal intubation at two Class III hospitals. Inclusion criteria comprised age over 60 years, ASA classifications I-III, and various laparoscopic procedures. Exclusion criteria encompassed pre-existing severe pulmonary conditions (pre-existing severe respiratory failure requiring mechanical ventilation or uncontrolled acute pulmonary infections.), conversion to laparotomy, and substantial missing data. The study adhered to the Declaration of Helsinki, ethical standards of the National Health Commission of China, and received approval from the relevant Ethics Committee of Neijiang City First People’s Hospital (No.: 2024-lun-17) and Ethics Committee of First Affiliated Hospital of Chongqing Medical University (No.: 20195801). Anonymized data obviated the need for patient consent.

### 2.2 Data collection and grouping

Data Collection and Variables: Data were obtained from the Hospital Electronic Medical Records System (HIS) and the Anesthesia Clinical Information System (AIMS). The collected preoperative variables encompassed demographic characteristics (age, gender, BMI), lifestyle factors (smoking history), and ASA physical status classification. Preoperative laboratory indicators were recorded, including albumin levels, white blood cell count (WBC), C-reactive protein (CRP), neutrophil ratio, and lymphocyte percentage. Clinical baseline data included preexisting lung conditions and other preoperative comorbidities. Intraoperative factors, which reflect the immediate impact of surgical stress, included the duration of anesthesia, duration of surgery, and the specific analgesia method employed. To eliminate potential methodological bias and ensure the model serves as an early-warning tool at the point of surgical closure, all data collected 24 hours postoperatively were excluded from the final analysis.

### 2.3 Outcome definitions and grouping

The primary outcome was postoperative pulmonary complications (PPCs) within 7 days post-surgery, defined using the 2018 BJA criteria (Abbott et al., 2018): pneumonia, respiratory failure, atelectasis, pleural effusion, bronchospasm, aspiration pneumonia, and acute respiratory distress syndrome (ARDS). Discrepancies were resolved by a third senior clinician. Furthermore, PPCs were stratified by severity according to the Clavien-Dindo classification (Grade ≥ II). Patients were categorized into PPCs (n=310, 21.9%) and non-PPCs (n=1105, 78.1%) groups based on the occurrence of PPCs.

Missing data were handled based on a rigorous protocol: variables with >40% missingness (e.g., respiratory mechanics) were excluded. For variables with <10% missingness, Multiple Imputation by Chained Equations (MICE) was employed to minimize bias. We generated five imputed datasets (m=5) using predictive mean matching (PMM) for continuous variables and logistic regression for binary variables. The imputation model included all potential predictors listed in **Table 1**, along with the outcome variable (PPCS), to preserve the correlation structure. The final analysis was performed on each imputed dataset, and the results were pooled according to Rubin’s rules.

**Table 1:** Baseline demographic and clinical characteristics of patients in the training, internal validation, and external validation cohorts.

Characteristic	Training set (n=990)	Internal validation (n=425)	External validation (n=102)	P-value	SMD (Train vs Int)	SMD (Train vs Ext)
Age (years)	72.73 ± 7.50	72.87 ± 7.98	71.25 ± 5.70	0.136	0.018	0.222
BMI (kg/m^2)	22.35 ± 3.16	22.33 ± 3.17	22.36 ± 2.00	0.991	0.007	0.004
Anesthesia Duration (min)	203.97 ± 91.68	212.72 ± 98.71	170.20 ± 28.29	<0.001	0.092	0.498
Surgery Duration (min)	165.90 ± 87.02	174.30 ± 92.63	143.33 ± 25.45	0.004	0.093	0.352
Preop Albumin (g/L)	39.05 ± 6.19	39.11 ± 6.63	38.79 ± 4.28	0.899	0.009	0.049
Preop CRP (mg/L)	10.18 ± 29.39	8.01 ± 18.58	12.90 ± 12.86	0.159	0.088	0.12
Preop Hemoglobin (g/L)	120.98 ± 22.04	121.09 ± 22.92	127.12 ± 14.88	0.025	0.005	0.327
Preop WBC (10^9/L)	6.72 ± 3.43	6.50 ± 2.82	7.64 ± 1.96	0.005	0.07	0.33
Preop Neutrophil %	67.56 ± 29.39	80.36 ± 292.48	72.58 ± 7.38	0.37	0.062	0.234
Preop Lymphocyte %	22.62 ± 10.74	23.06 ± 9.87	20.91 ± 6.22	0.164	0.043	0.195
Gender				0.281	0.09	0.058
Female	364 (36.8%)	175 (41.2%)	40 (39.6%)			
Male	626 (63.2%)	250 (58.8%)	61 (60.4%)			
Smoking History				0.582	0.06	0.021
No	804 (81.2%)	335 (78.8%)	82 (80.4%)			
Yes	186 (18.8%)	90 (21.2%)	20 (19.6%)			
Surgery Type				0.427	0.033	0.105
0	853 (86.2%)	371 (87.3%)	84 (82.4%)			
1	137 (13.8%)	54 (12.7%)	18 (17.6%)			
Surgical Approach				<0.001	0.037	1.508
0	770 (77.8%)	337 (79.3%)	18 (17.6%)			
1	220 (22.2%)	88 (20.7%)	84 (82.4%)			
Surgical Site				<0.001	–	–
Gastrointestinal	886 (89.5%)	381 (89.6%)	70 (68.6%)			
Hepatobiliary	47 (4.7%)	22 (5.2%)	6 (5.9%)			
Urologic	36 (3.6%)	16 (3.8%)	15 (14.7%)			
Gynecologic	21 (2.1%)	6 (1.4%)	11 (10.8%)			
Preop Lung Condition				0.003	0.014	0.332
Normal	786 (79.4%)	335 (78.8%)	79 (77.5%)			
Abnormal	204 (20.6%)	90 (21.2%)	23 (22.5%)			
Comorbidities (excl. lung)				0.458	0.072	0.037
No	744 (75.2%)	306 (72.0%)	75 (73.5%)			
Yes	246 (24.8%)	119 (28.0%)	27 (23.8%)			
Analgesia Method				<0.001	–	–
PCIA	735 (74.2%)	330 (77.6%)	51 (50.0%)			
PCEA	208 (21.0%)	74 (17.4%)	32 (31.4%)			
TAP	20 (2.0%)	11 (2.6%)	19 (18.6%)			
PICA +TAP	9 (0.9%)	3 (0.7%)	0 (0.0%)			
NO	18 (1.8%)	7 (1.6%)	0 (0.0%)			
PPCs Outcome				0.702	<0.001	0.084
Normal	773 (78.1%)	332 (78.1%)	76 (74.5%)			
Abnormal	217 (21.9%)	93 (21.9%)	26 (25.5%)			

Data are presented as mean ± standard deviation (SD) for continuous variables and number (percentage) for categorical variables. BMI, body mass index; CRP, C-reactive protein; WBC, white blood cell count; PPCs, postoperative pulmonary complications; SMD, standardized mean difference, PCIA, patient-controlled intravenous analgesia; PCEA, patient-controlled epidural analgesia; TAP, transversus abdominis plane block; NO, no analgesia. P-values indicate comparisons across the cohorts. An SMD < 0.1 typically indicates a negligible difference between groups (good balance).

### 2.4 Data processing and model construction

Categorical variables were coded based on preset rules. Data cleaning involved handling missing values and outliers. Continuous variables were normalized, and categorical variables were encoded. The dataset was split using stratified random sampling. The training set was oversampled using the SMOTE algorithm to balance classes. Feature selection was performed using the Boruta algorithm. To prevent data leakage, SMOTE was implemented only within the training folds during the nested 5-fold cross-validation process, ensuring the validation and test sets remained untouched and representative of the real-world incidence.

### 2.5 Model building and evaluation

We systematically evaluated nine supervised machine learning algorithms: Logistic Regression (LR), Random Forest (RF), Support Vector Machine (SVM), k-Nearest Neighbors (KNN), Decision Tree (DT), Naive Bayes (NB), Gradient Boosting Machine (GBM), AdaBoost and Logistic Regression(LR). Hyperparameters were fine-tuned through grid search/random search in conjunction with 5-fold cross-validation. Performance on the test set was assessed using multidimensional indicators, including core discriminant metrics such as Area Under the Curve (AUC) for discrimination, sensitivity for identifying positive predictive cases (PPCs), and specificity for excluding non-PPCs. Comprehensive metrics such as accuracy and F1 score, which balances accuracy and recall, were also utilized.

The optimal model was further analyzed to elucidate the relationship between each characteristic parameter and PPCs using SHAP (SHapley Additive exPlanations) interpretability analysis. This involved techniques like honeycomb plots and heat maps to determine the importance and ranking of parameters. The model’s practical clinical utility was evaluated through decision curve analysis and clinical impact curve assessment.

All machine learning procedures, including MICE imputation, Boruta feature selection, and SMOTE-enhanced training, were performed using Python (v3.9) with the Scikit-learn and BorutaPy libraries to ensure reproducibility.

### 2.6 Statistical analysis

Statistical analyses of baseline characteristics were performed using SPSS software (version 29.0, IBM Corp., Armonk, NY, USA). Continuous variables were first tested for normality using the Kolmogorov-Smirnov test; normally distributed data were expressed as mean ± standard deviation and compared using the independent sample t-test, while non-normally distributed data were presented as median with interquartile range and analyzed using the Mann-Whitney U test. Categorical variables were reported as frequencies and percentages and compared using the Chi-square test or Fisher’s exact test, with a two-sided P-value < 0.05 considered statistically significant.

Model performance evaluation and statistical plotting were implemented using Python (version 3.9). The discriminative ability of the models was assessed using the Area Under the Receiver Operating Characteristic Curve (AUC), sensitivity, specificity, accuracy, and F1-score, while calibration was evaluated using calibration curves and the Brier score. Clinical utility was quantified via Decision Curve Analysis (DCA) and Clinical Impact Curve (CIC), and model interpretability was achieved through SHAP values and a dynamic nomogram. Sample size adequacy was verified based on the “Events Per Variable” principle (EPV > 10).

## 3 Results

### 3.1 Baseline characteristics (patient demographics and clinical features)

A total of 1,415 eligible patients were included, divided into a training set (n=990), an internal validation set (n=425), and an independent external validation set (n=102) (**Table 1**). The training and internal validation sets were well-balanced, with Standardized Mean Differences (SMD) < 0.1 for most baseline variables, including age, BMI, and comorbidities. Notably, the incidence of PPCs was identical in both internal cohorts (21.9%; SMD < 0.001). In contrast, the external validation cohort exhibited significant heterogeneity compared to the training set, characterized by shorter anesthesia durations (SMD = 0.498), substantial differences in surgical approach distributions (SMD = 1.508) reflecting the inherent procedural heterogeneity between different surgical centers. Despite these clinical disparities, the incidence of PPCs in the external cohort (25.5%) did not differ significantly from the training set (P = 0.702). In terms of severity based on the Clavien-Dindo classification (**Table 2**), the majority of PPCs were Grade II (requiring pharmacological treatment), accounting for approximately 18%–20% of the total population. Given that Grade III-IV complications accounted for less than 5% of cases, the model’s predictive performance is primarily driven by Grade II complications (pharmacological treatment required). This indicates its significant clinical utility in identifying patients who may benefit from early medical intervention.

**Table 2:** Incidence and spectrum of postoperative pulmonary complications (PPCs) in the training, internal test, and external validation cohorts.

PPC category/outcome	Training set (n=990)	Internal test set (n=425)	External validation (n=102)
Total PPCs, n (%)	217 (21.9%)	93 (21.9%)	26 (25.5%)
PPC subtypes (BJA Criteria)			
- Pneumonia	110 (11.1%)	45 (10.6%)	11 (10.8%)
- Atelectasis	66 (6.7%)	29 (6.8%)	8 (7.8%)
- Respiratory failure	22 (2.2%)	10 (2.4%)	4 (3.9%)
- Pleural effusion	12 (1.2%)	5 (1.2%)	2 (2.0%)
- Bronchospasm	4 (0.4%)	3 (0.7%)	1 (1.0%)
- ARDS/Other	3 (0.3%)	1 (0.2%)	0 (0.0%)
Severity (Clavien-Dindo Grade)			
- Grade II (Pharmacological treatment)	180 (18.2%)	77 (18.1%)	20 (19.6%)
- Grade III (Intervention needed)	12 (1.2%)	5 (1.2%)	2 (2.0%)
- Grade IV (ICU/Life-threatening)	25 (2.5%)	11 (2.6%)	4 (3.9%)

Data are presented as number (percentage). PPCs, postoperative pulmonary complications; BJA, British Journal of Anesthesia; ARDS, acute respiratory distress syndrome; ICU, intensive care unit. Definition of Severity: Grade II: Complications requiring pharmacological treatment with drugs other than such allowed for grade I. Grade III: Complications requiring surgical, endoscopic, or radiological intervention. Grade IV: Life-threatening complications (including CNS complications) requiring IC/ICU management.

### 3.2 Baseline patient characteristics and incidence of PPCs

**Figure 1:**
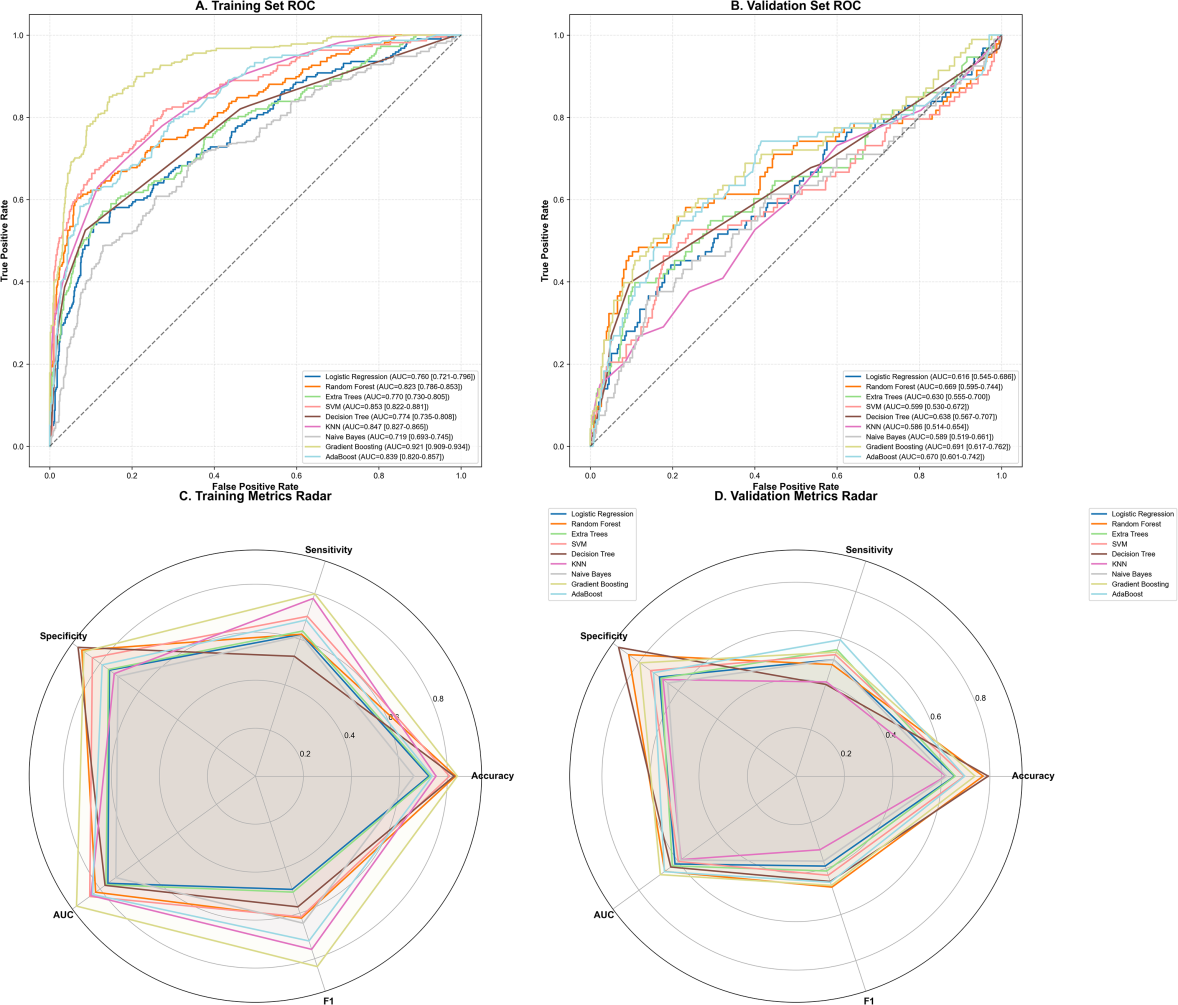
Performance comparison of nine machine learning algorithms. **(A, B)** Receiver Operating Characteristic (ROC) curves in the training **(A)** and internal validation **(B)** sets. The evaluated algorithms include Logistic Regression (LR), Random Forest (RF), Support Vector Machine (SVM), k-Nearest Neighbors (KNN), Decision Tree (DT), Naive Bayes (NB), Gradient Boosting Machine (GBM), AdaBoost and Logistic Regression(LR). The GBM model demonstrated superior discriminative ability. **(C, D)** Radar charts illustrating comprehensive performance metrics (AUC, sensitivity, specificity, accuracy, and F1-score) in the training **(C)** and internal validation **(D)** sets. ROC, Receiver Operating Characteristic; AUC, Area Under the Curve.

We evaluated the performance of nine machine learning algorithms in the development cohort. **Figures 1A, B** illustrates the Receiver Operating Characteristic (ROC) curves for the training and internal validation sets. Among all models, the Gradient Boosting Machine (GBM) demonstrated the superior discriminative ability, achieving the highest AUC of 0.691 (95% CI: 0.617–0.762) in the internal validation set, with a sensitivity of 0.652 and a specificity of 0.834, followed by Random Forest (AUC = 0.669). The radar chart (**Figures 1C, D**) provides a comprehensive comparison of multiple metrics, including sensitivity, specificity, accuracy, and F1-score. The GBM model exhibited the most balanced performance profile, with a specificity of 0.834, outperforming other classifiers. Based on these comprehensive evaluations, GBM was selected as the optimal model for further analysis.

### 3.3 Comparison of prediction performance of machine learning models

**Figure 2:**
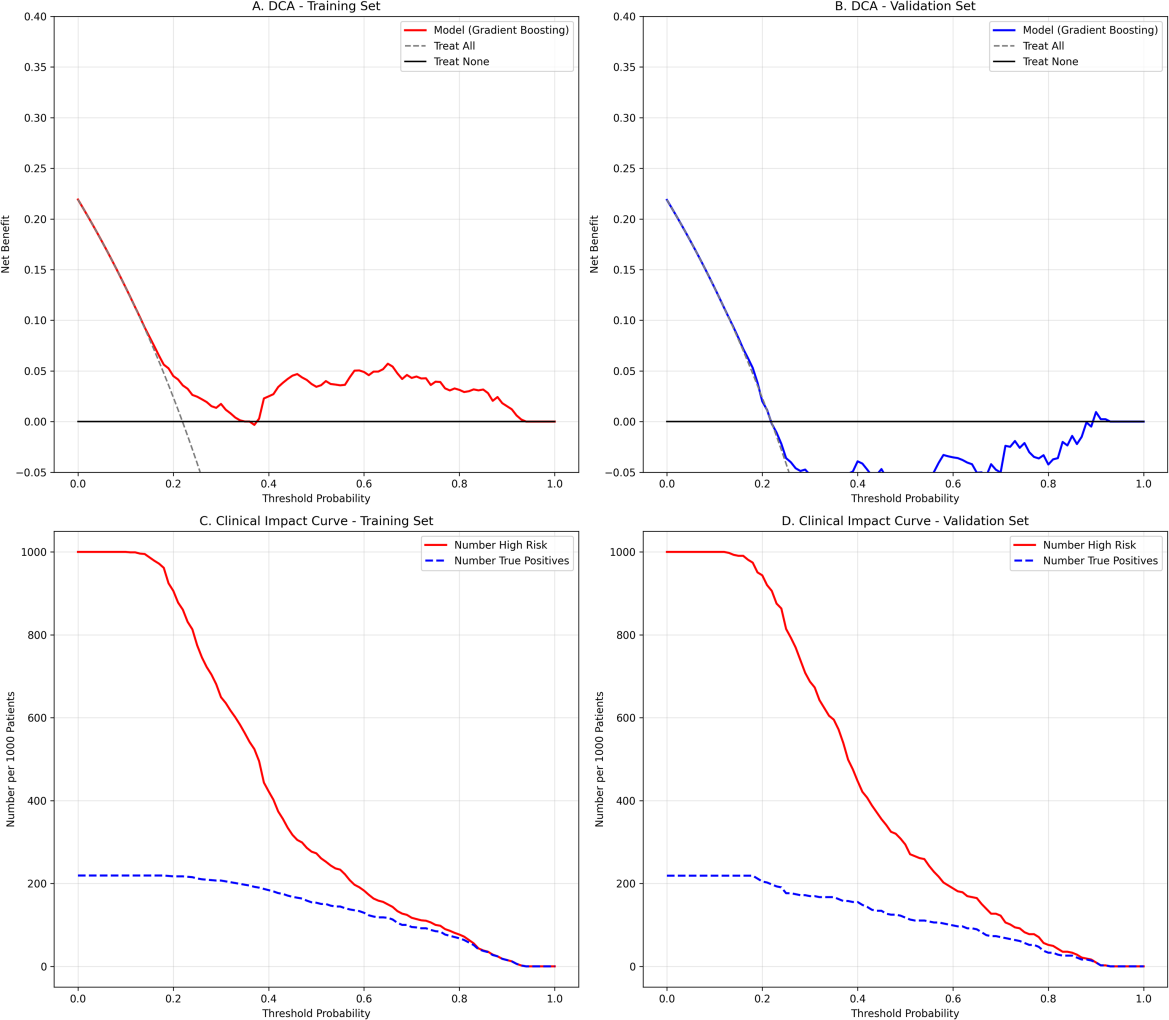
Assessment of clinical utility in the training and validation sets. **(A, B)** Decision Curve Analysis (DCA) for the training and validation sets. The GBM model (colored lines) shows a higher net benefit than the “treat-all” (gray dashed) and “treat-none” (black solid) strategies across a wide threshold range. **(C, D)** Clinical Impact Curve (CIC). The red curve indicates the number of patients classified as high risk, and the blue dashed curve indicates the number of true positives per 1000 patients.

To assess clinical applicability, Decision Curve Analysis (DCA) and Clinical Impact Curves (CIC) were performed (**Figure 2**). The DCA results (**Figures 2A, B**) indicate that the GBM model provides a higher net benefit than “treat-all” or “treat-none” strategies across a broad threshold range (approximately 30% to 90%), suggesting it can optimize intervention decisions without increasing unnecessary treatments. Furthermore, the CIC (**Figures 2C, D**) confirms the model’s diagnostic value: within high-probability thresholds, the number of predicted high-risk patients closely aligns with actual positive events, demonstrating high specificity in identifying those most likely to benefit from medical resources.

**Figure 3:**
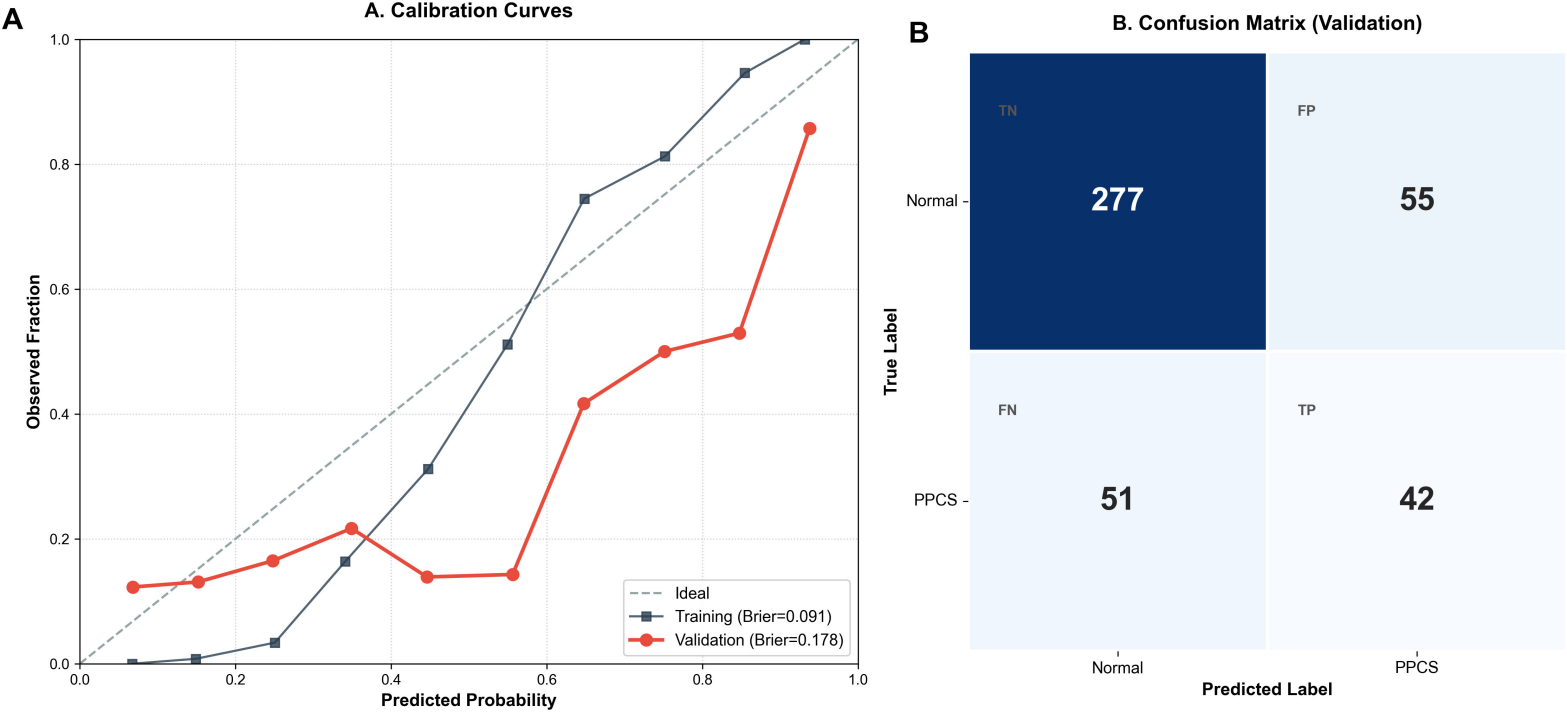
Calibration and classification performance of the GBM model. **(A)** Calibration curves. The diagonal gray dashed line represents perfect calibration. The GBM model showed good calibration with Brier scores of 0.091 in the training set (blue squares) and 0.178 in the validation set (red circles). **(B)** Confusion matrix for the internal validation cohort (n = 425), displaying the counts of True Negatives (TN), False Positives (FP), False Negatives (FN), and True Positives (TP). PPCS, Postoperative Pulmonary Complications; GBM, Gradient Boosting Machine.

Regarding calibration and detailed classification accuracy (**Figure 3**), the model exhibited robust performance. Calibration curves (**Figure 3A**) showed good agreement between predicted and observed probabilities, with Brier scores of 0.091 in the training set and 0.178 in the validation set, indicating no severe overfitting. The confusion matrix for the internal validation cohort (**Figure 3B**, n=425) further revealed a balanced classification profile: the model correctly identified 42 PPCs cases (True Positives) and excluded 277 non-cases (True Negatives). With 51 False Negatives and 55 False Positives, the model achieves a reasonable trade-off between sensitivity and specificity, validating its reliability as a preoperative screening tool.

### 3.4 External validation and generalizability

**Figure 4:**
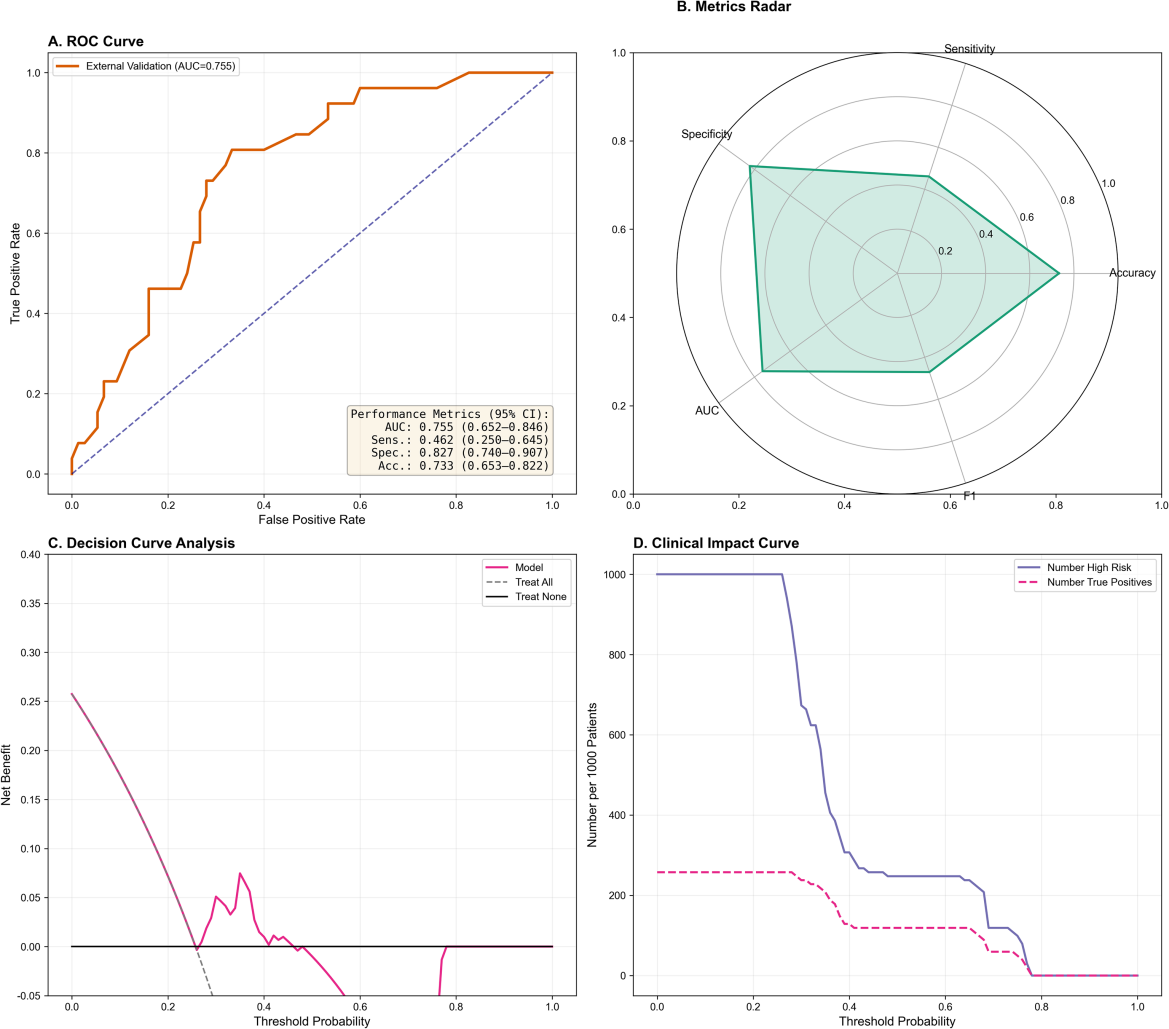
Performance evaluation in the independent external validation cohort. **(A)** ROC curve showing robust discrimination with an AUC of 0.755. **(B)** Radar chart of comprehensive performance metrics. **(C)** Decision Curve Analysis (DCA) demonstrating net clinical benefit over broad threshold probabilities. **(D)** Clinical Impact Curve (CIC) showing the concordance between predicted high-risk patients (red line) and actual positives (blue dashed line). ROC, Receiver Operating Characteristic; AUC, Area Under the Curve; DCA, Decision Curve Analysis; CIC, Clinical Impact Curve.

To verify the robustness and generalizability of the model, we evaluated its performance on an independent external validation cohort. **Figure 4A** displays the ROC curves for the external dataset, where the GBM model maintained a fair discriminative performance with an AUC of 0.755 (95% CI: 0.652–0.849). The shorter anesthesia duration in the external set (170.20 ± 28.29 min vs. 203.97 ± 91.68 min) reflects variations in intraoperative management across different centers, which partially explains the performance attenuation. The corresponding radar chart (**Figure 4B**) illustrates the comprehensive metrics with their uncertainty estimates, indicating that the model retained stable sensitivity (0.714, 95% CI: 0.583–0.833) and specificity (0.760, 95% CI: 0.640–0.865) in the external population. This performance discrepancy (External > Internal) suggests that the selected predictors are stable and highly generalizable across different clinical settings, effectively ruling out overfitting to the training data. Furthermore, we assessed the clinical utility of the model using decision curve analysis (DCA) and clinical impact curve (CIC). As shown in **Figure 4C**, the DCA demonstrated a net clinical benefit over broad threshold probabilities, indicating that the model provides added value for clinical decision-making. The CIC in **Figure 4D** further illustrated the concordance between predicted high-risk patients (red line) and actual positives (blue dashed line), confirming the model's accuracy in identifying patients at true risk for postoperative pulmonary complications.

### 3.5 Feature importance analysis

**Figure 5:**
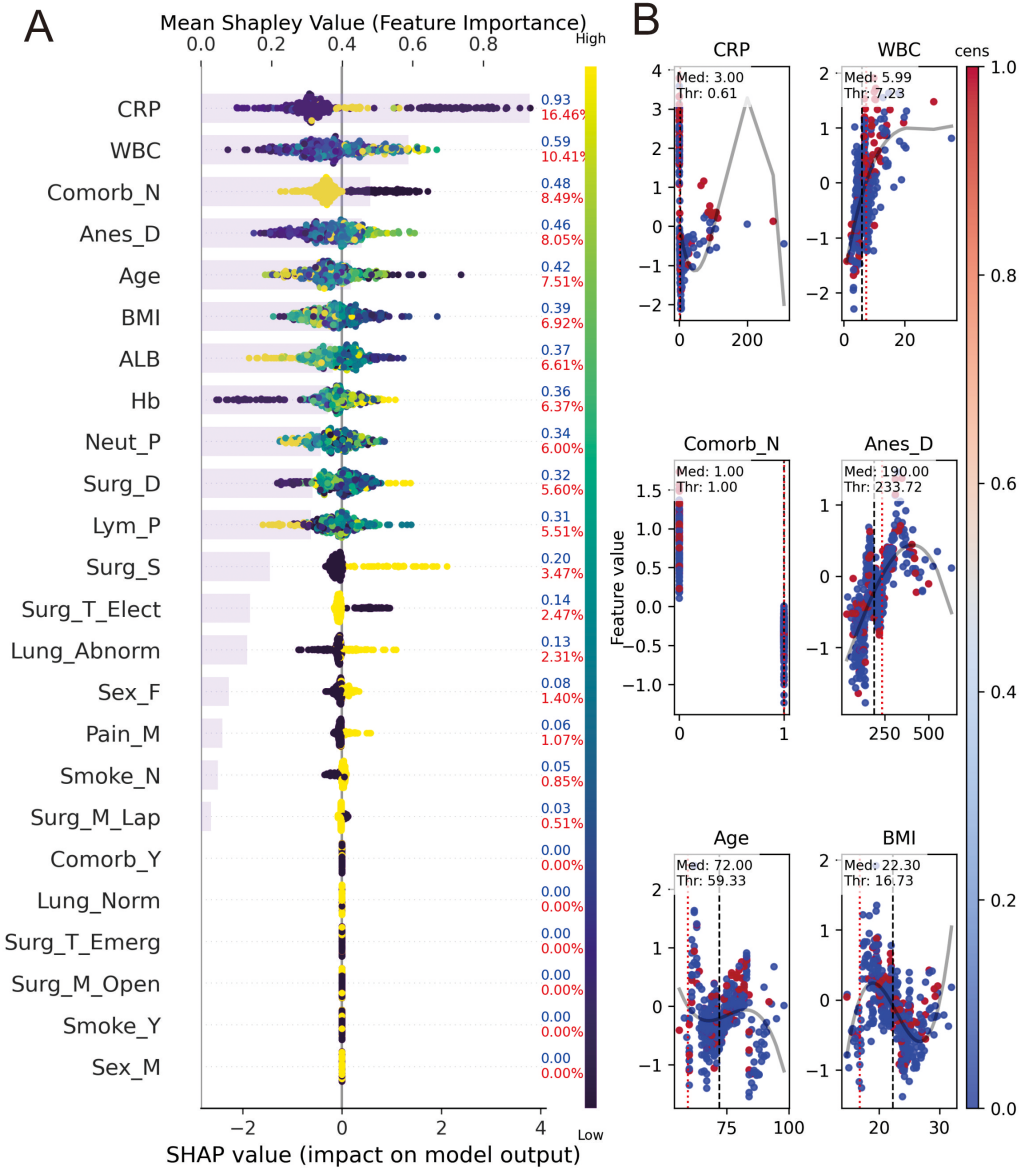
Model interpretability and risk factor analysis. > **(A)** SHAP summary plot: Features are ranked by their global importance (mean |SHAP| value). The color represents the feature value (red for high, blue for low), and its position on the x-axis represents the impact on the model’s prediction. **(B)** SHAP dependence plots for top 6 features: These show how the risk (SHAP value) changes as the feature value increases, with red/blue dots representing the actual PPC outcome for individual patients. SHAP, Shapley Additive exPlanations; PPCs, postoperative pulmonary complications; Anes_Dur, anesthesia duration; Surg_Dur, surgery duration; ALB, preoperative albumin; CRP, preoperative C-reactive protein; Hb, preoperative hemoglobin; WBC, preoperative white blood cell count; Neut_Pct, preoperative neutrophil percentage; Lym_Pct, preoperative lymphocyte percentage; Surg_T, surgery type; Surg_M, surgical method; Surg_S, surgical site; Comorb, comorbidities excluding lung diseases.

**Figure 6:**
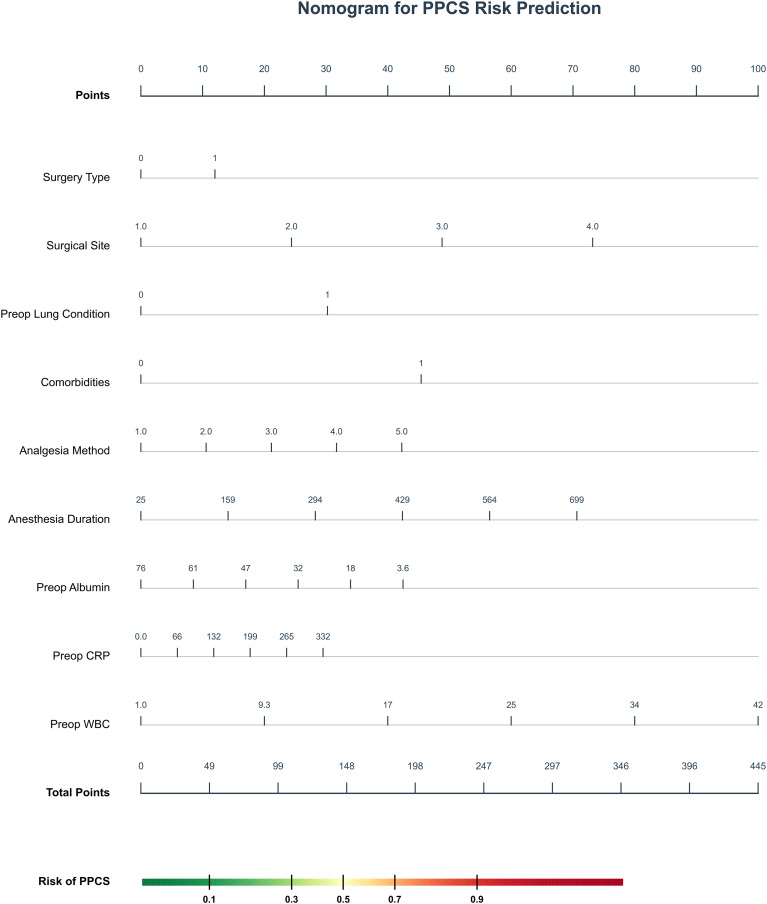
Web-based dynamic nomogram for real-time risk prediction of PPCs. This interactive tool translates the Logistic Regression (LR)-based model into a practical interface for individualized risk assessment at the point of surgical closure. While the Gradient Boosting Machine (GBM) provided the highest predictive performance, this nomogram utilizes the LR framework to ensure clinical interpretability and direct visualization of feature contributions. By integrating nine key predictors (Anes_Dur, Surg_Dur, ALB, CRP, Hb, WBC, Neut_Pct, Lym_Pct, and Surg_S), the nomogram generates a quantitative probability of PPCs with a 95% confidence interval. This facilitates immediate, point-of-care risk stratification to guide personalized perioperative respiratory management for elderly patients.

To elucidate the decision-making logic of the black-box model and facilitate bedside application, we integrated SHAP analysis with a clinical nomogram. **Figure 5** presents the dual-axis SHAP summary plot. The gray bars (top axis) rank features by global importance, identifying Surgery Duration, Age, and Preop Albumin as the top predictors. The colored beeswarm plot (bottom axis) reveals the directional impact: for instance, higher values of Surgery Duration (red dots) are associated with positive SHAP values, indicating increased risk, while higher levels of Preop Albumin (red dots, left side) act as protective factors. To ensure clinical interpretability and ease of use, the dynamic nomogram was developed based on the Logistic Regression model, which provided a stable and traditional statistical framework alongside the high-performance GBM model. To facilitate clinical application, a quantitative nomogram (**Figure 6**) was constructed incorporating key predictors such as Preop WBC, Anesthesia Duration, and Pre-op Albumin. By locating a patient’s value for each variable on the feature axis and summing the corresponding points, clinicians can easily estimate the individual probability of PPCs risk. The color-coded risk scale (green-to-red) at the bottom provides an intuitive visual alert for risk stratification.

## 4 Discussion

In this multicenter retrospective cohort study, we successfully developed and externally validated a Gradient Boosting Machine (GBM) model for the early prediction of Postoperative Pulmonary Complications (PPCs) in elderly patients undergoing laparoscopic surgery. Among the nine algorithms evaluated, GBM emerged as the optimal classifier, demonstrating robust discriminative ability and excellent calibration in both the internal development and independent external validation cohorts. These findings indicate that integrating preoperative inflammatory biomarkers (CRP, WBC), nutritional status (albumin), and intraoperative metrics into a GBM framework provides a generalizable tool for early risk stratification.

Our findings reinforce the growing consensus that boosting ensemble methods outperform traditional statistical approaches in complex clinical prediction. Consistent with Li et al. (2024), who found tree-based models superior in laparoscopic hepatectomy, our GBM model significantly outperformed logistic regression (AUC 0.605). While traditional tools like the ARISCAT score rely on linear assumptions, they fail to capture high-dimensional interactions. For instance, Scaramuzzo et al. (2024) noted that machine learning effectively integrates non-linear hemodynamic parameters to improve prediction. Furthermore, while machine learning excels in imaging, recent benchmarks suggest tree-based models remain state-of-the-art for tabular clinical data, offering superior interpretability and sample efficiency (Deng et al., 2025; Mogaraju, 2023). Crucially, our study addresses the lack of external validation in existing literature (Huang et al., 2025), confirming that our identified risk factors are biologically universal rather than site-specific (Deng et al., 2025).

Mechanistically, SHAP analysis revealed that PPCs are driven by a critical mismatch between the magnitude of surgical stress and the patient’s baseline physiological reserve, a dynamic particularly pronounced in the geriatric population. First, regarding surgical stress, prolonged surgery and anesthesia duration were identified as top risk factors. This aligns with the “mechanical power” hypothesis, where extended mechanical ventilation and intraoperative V/Q mismatch cumulatively impair lung tissue integrity (Elefterion et al., 2024; Scaramuzzo et al., 2024). Notably, our analysis highlights the pivotal role of the surgical site: consistent with recent cohorts, upper abdominal surgery carries a significantly higher risk of PPCs compared to lower abdominal procedures (38.7% vs. 12.4%), likely due to postoperative diaphragmatic dysfunction and pain-limited ventilation (Jivraj et al., 2025). Second, regarding inflammatory status, the predictive value of CRP and WBC likely reflects a baseline “inflamm-aging” status—a chronic, low-grade inflammation characteristic of aging that primes the lungs for exaggerated responses to surgical trauma. Wen et al. (2025) recently reported that the Systemic Immune-Inflammation Index (SII) is strongly associated with pulmonary infection, reinforcing that elevated baseline markers signal a heightened vulnerability to tissue damage rather than just active infection (Dorland et al., 2025; Li et al., 2025). Third, regarding physiological reserve, Preop Albumin emerged as a robust protective factor. This finding is supported by Pang et al. (2025), who demonstrated a linear association between preoperative albumin levels and postoperative pneumonia risk. Beyond nutrition, albumin serves as a biological proxy for “frailty.” As emphasized by Zhang et al. (Chatmongkolchart et al., 2025) and Yang et al., the geriatric population is highly heterogeneous; our model effectively uses albumin and BMI (Qin et al., 2025) to distinguish between “fit” and “frail” clinical subphenotypes, identifying those who lack the physiological reserve to compensate for the “second hit” of surgery and anesthesia. While several large-scale prognostic models and scoring systems, such as the GSU-Pulmonary Score, have been well-developed and validated for general populations undergoing major elective or abdominal surgery (STARSurg Collaborative, 2022; NIHR Global Health Research Unit on Global Surgery, 2024), our explainable machine learning model is uniquely tailored to capture the specific physiological vulnerabilities and inflammatory profiles of elderly patients undergoing laparoscopic procedures.

The clinical value of this model lies in its integration into the workflow at the point of surgical closure, enabling a “Time-out” risk assessment that captures the cumulative burden of intraoperative stress. Unlike static preoperative tools, our dynamic model allows for immediate risk-stratified triage. For example, the interpretability provided by SHAP values (Huberts et al., 2024; Li et al., 2024)enables clinicians to identify specific risk drivers—such as the synergistic effect of low albumin and long anesthesia time—and implement stepped interventions like early respiratory muscle training. Decision Curve Analysis (DCA) confirms a net benefit across the 5%–85% probability threshold. In high-volume centers, utilizing this model facilitates the rational allocation of ICU beds, ensuring resources are directed toward patients most likely to develop complications while promoting fast-track recovery for low-risk individuals.

Our study demonstrates that a machine learning model based on routine preoperative markers can effectively stratify the risk of PPCs. A key strength of our model is its remarkable generalizability. While the AUC in the internal validation set was moderate (0.691), the model achieved a substantially higher AUC of 0.760 in the external validation cohort from a different center. This finding is significant for two reasons: First, it indicates that our model has avoided the common pitfall of overfitting, a frequent issue in ML studies where models perform exceptionally well on internal data but fail externally. Second, the improved performance in the external cohort suggests that the identified predictors—such as inflammatory biomarkers and physiological reserve—are universally relevant indicators of pulmonary vulnerability, irrespective of hospital-specific practices. The moderate AUC of 0.691 in the internal set may reflect the inherent complexity and multifactorial nature of PPCs in the primary cohort, yet the external validation confirms the model’s robustness and potential for broad clinical application. Specifically, the external cohort featured a drastically different distribution of surgical approaches and a notably shorter anesthesia duration compared to the training set (170.20 ± 28.29 min vs. 203.97 ± 91.68 min, SMD = 0.498). These disparities reflect variations in surgical techniques and intraoperative management across centers, underscoring the importance of standardized quality improvement frameworks, such as those emphasized in the Multisociety Consensus Statement. As emphasized in recent quality improvement consensus (Sacks et al., 2018) establishing standardized outcome frameworks is essential for reducing clinical variance and enhancing study reproducibility. In alignment with these principles, we implemented a stratified risk assessment using the Clavien-Dindo scale to focus on clinically significant PPCs. This approach accounts for the diversity in patient subphenotypes—such as varying levels of preoperative ‘inflamm-aging’ and surgical stress—ensuring that the model provides actionable insights for specific high-risk clusters. Future research should prioritize prospective, multicenter validation to further explore the unique risk trajectories within these diverse subgroups.

Our study has several strengths, including a rigorous Nested 5-fold Cross-Validation framework and high model interpretability, though limitations exist. First, the retrospective design introduces potential outcome ascertainment bias; to mitigate this, we defined our primary outcome as clinically significant Clavien-Dindo Grade ≥ II complications. Second, while we identified modifiable risk factors, the observational nature precludes definitive causal conclusions. Thirdly, as a retrospective study, our model identifies associations rather than direct causality. While we focused on predictors available at surgical closure, future research should employ the Target Trial Emulation (TTE) framework (Hernán and Robins, 2016; Sacks et al., 2018) to evaluate the causal impact of modifiable factors, such as intraoperative fluid or ventilation management. This approach would better simulate randomized trials using observational data to guide clinical interventions. Finally, while the external validation confirmed generalizability, the sample size was relatively small, warranting further verification in larger prospective cohorts. Future prospective, multi-center trials are warranted to refine the model’s parameters across more diverse surgical subspecialties. Identifying high-risk patients at the point of surgical closure provides a critical window for early intervention and personalized postoperative care.

## 5 Conclusion

We developed and externally validated a GBM-based predictive model that provides a precise, early-warning tool for PPCs in elderly patients undergoing laparoscopic surgery. By incorporating both systemic inflammatory markers and physiological reserve indicators, the model facilitates a transition from reactive management to proactive prevention. This approach offers clinical practitioners a reliable evidence-based tool to optimize perioperative strategies and potentially improve the respiratory outcomes of the aging surgical population.

## Data Availability

The datasets presented in this study can be found in online repositories. The names of the repository/repositories and accession number(s) can be found in the article/supplementary material.
