# Correction: Inflammatory biomarkers and physiological reserve: an explainable machine learning model for predicting postoperative pulmonary complications in elderly laparoscopic surgery

**DOI:** 10.3389/fcimb.2026.1835273

**Published:** 2026-04-08

**Authors:** Di Liu, Fei Jiang, Hui Huang, Yong Yang, Lei Zou, Min Ye

**Affiliations:** 1 Anesthesia Surgery Center, The First People’s Hospital of Neijiang, Neijiang, Sichuan, China; 2 Department of Radiology, The First People’s Hospital of Neijiang, Neijiang, Sichuan, China; 3 Department of Anesthesiology, The First Affiliated Hospital of Chongqing Medical University, Chongqing, China

**Keywords:** early warning, elderly, gradient boosting machine, laparoscopic surgery, machine learning, postoperative pulmonary complications

The order of authors in the author list of the published paper was erroneously given as: “Di Liu^1^, Fei Jiang^1^, Hui Huang^1^,Yong Yang ^1^, Min Ye^2*^,Lei Zou^3*^”. The correct author list is: “Di Liu^1^, Fei Jiang^1^, Hui Huang^1^,Yong Yang ^1^, Lei Zou^2*^, Min Ye^3*”^

Author Min Ye was erroneously assigned as the first corresponding author. The correct corresponding author structure should designate Lei Zou as the first corresponding author and Min Ye as the second (final) corresponding author.

The **Author Contributions** statement was erroneously given as: “DL: Conceptualization, Funding acquisition, Methodology, Project administration, Software, Visualization, Writing – original draft, Writing – review & editing. FJ: Data curation, Investigation, Resources, Software, Supervision, Validation, Writing – original draft. HH: Formal analysis, Funding acquisition, Investigation, Project administration, Software, Supervision, Visualization, Writing – original draft. YY: Data curation, Formal analysis, Funding acquisition, Resources, Supervision, Visualization, Writing – original draft. MY: Formal analysis, Funding acquisition, Investigation, Methodology, Project administration, Resources, Software, Writing – original draft, Writing – review & editing. LZ: Data curation, Formal analysis, Investigation, Project administration, Supervision, Visualization, Writing – review & editing.”

The correct **Author Contributions** statement is: “DL: Conceptualization, Funding acquisition, Methodology, Project administration, Software, Visualization, Writing – original draft, Writing – review & editing. FJ: Data curation, Investigation, Resources, Software, Supervision, Validation, Writing – original draft. HH: Formal analysis, Funding acquisition, Investigation, Project administration, Software, Supervision, Visualization, Writing – original draft. YY: Data curation, Formal analysis, Funding acquisition, Resources, Supervision, Visualization, Writing – original draft. LZ: Data curation, Formal analysis, Investigation, Project administration, Supervision, Visualization, Writing – review & editing. MY: Formal analysis, Funding acquisition, Investigation, Methodology, Project administration, Resources, Software, Writing – original draft, Writing – review & editing.”

